# Enhancing Physical and Psychosocial Health of Older Adults in Saudi Arabia through Walking: Comparison between Supervised Group-Based and Non-Supervised Individual-Based Walking

**DOI:** 10.3390/ejihpe13110165

**Published:** 2023-10-26

**Authors:** Palash Karmakar, Ming-Yu (Claudia) Wong, Mezna A. AlMarzooqi, Nouf Alghamdi, Kailing Ou, Yanping Duan, Ryan E. Rhodes, Chun-Qing Zhang

**Affiliations:** 1 Department of Sport, Physical Education and Health, Hong Kong Baptist University, Hong Kong, China; 19481241@life.hkbu.edu.hk (P.K.); 21482268@life.hkbu.edu.hk (K.O.); duanyp@hkbu.edu.hk (Y.D.); 2 Department of Pharmacy, Noakhali Science and Technology University, Noakhali 3814, Bangladesh; 3 Department of Health and Physical Education, The Education University of Hong Kong, Hong Kong, China; 4 Department of Community Health Sciences, College of Applied Medical Science, King Saud University, Riyadh 11451, Saudi Arabia; malmarzooqi@ksu.edu.sa (M.A.A.); nalgamdi@ksu.edu.sa (N.A.); 5 Leaders Development Institute, Ministry of Sport, Riyadh 12641, Saudi Arabia; 6 School of Exercise Science, Physical & Health Education, University of Victoria, Victoria, BC V8P 5C2, Canada; rhodes@uvic.ca; 7 Department of Psychology, Sun Yat-sen University, Guangzhou 510275, China; zhangchq28@mail.sysu.edu.cn

**Keywords:** walking intervention, older adults, frailty syndrome, cognitive skill, quality of life, Saudi Arabia

## Abstract

Walking is widely recognized as one of the most common and effective forms of physical activity, particularly for older adults. This study examined the comparative effects of two types of walking interventions, including supervised group-based intervention (SGBI) and non-supervised individual-based intervention (NSIBI), on frailty syndrome, cognitive functions or skills, and health-related quality of life among sedentary older Saudi individuals. A 15-week double-blinded, randomized controlled trial (RCT) including three groups (two were intervention groups while the other was the control group) was conducted among older adults who were inactive to examine the effect of different forms of walking interventions on frailty syndrome, cognitive functions, and health-related quality of life. A total of 107 participants, including 65 males and 42 females, were divided into three groups, which include SGBI, NSIBI, and the control group. Frailty syndrome was measured using the physical performance test (PPT), while cognitive function and health-related quality of life were assessed using the Mini-mental State Examination (MMSE) and the Short Form 36 (SF 36) health survey questionnaire. One-way repeated measures analysis of variance (ANOVA) and one-way analysis of covariance (ANCOVA) with the pre- and post-tests were performed for within- and between-group differences. while post-test data for the control group participants were absent due to the change in elderly center regulation, and they were excluded from the analysis. Hence, the comparison was stated only between the intervention groups. Both of the intervention groups (SGBI and NSIBI) showed significant within-subject differences in the Physical Function subscale of the health-related quality of life scale only, with F(1,20) = 23.03, *p* < 0.001, and F(1,18) = 27.22, *p* < 0.001, respectively. On the other hand, the Physical Performance Test revealed significant [F(2,51) = 9.21, *p* < 0.001] between-group differences in the post-test based on the baseline values. In addition, the average step count of older adults was increased from 4000 steps per session to around 7000 steps per session in the intervention group. The average heart rate of the NSIBI group did not show a visible change, and the resting heart rate of both groups showed a slightly declining trend throughout the intervention period. The walking intervention significantly increased participants’ physical function, which is a component of health-related quality of life and physical performance (frailty level), along with average daily step counts for older adults in Saudi Arabia. Regular engagement in the recommended level of walking is strongly advisable for Saudi Arabian older adults to maintain their overall quality of life at this stage.

## 1. Background

Worldwide, older adults are considered the fastest-growing age group [1]. Globally, in 2020, the estimated number of people aged 65 years and older was 727 million, and it is projected to increase by over two-fold by 2050, with one out of every six people in the world being older adults [2,3]. Because of the aging process, older adults usually lead a sedentary life that can increase the risk of several health complications, which include excessive weight gain, cardiovascular diseases, different forms of bone disorders, diabetes, frailty, declined cognitive functions, impaired overall quality of life, and even mortality [4,5,6]. Furthermore, older individuals are more prone to psychological health issues, particularly depression [7], as well as experiencing feelings of loneliness and insufficient social support [8,9].

In line with the global trend, the Kingdom of Saudi Arabia (KSA) has also experienced an increased number of older adults due to the better improvement in medical science and consciousness regarding well-being, as well as the advancement in the healthcare sector and living standards. In 2019, the percentage of older adults aged 65 years and older was 2.4% of the total population [10] and is predicted to increase to 18.4% in 2050 [11]. In Saudi Arabia, the prevalence of people who are physically inactive is higher, as evidenced by a study that reported that 60% of middle-aged and older adults were physically inactive [12]. Furthermore, age-related complications, including osteoporosis, diabetes, hypertension, asthma, excessive weight gain, stroke, dementia, and diseases of the kidney and heart, are more widespread among older adults in the Kingdom of Saudi Arabia [13]. Evidence revealed that, due to the higher prevalence of chronic diseases, the growing number of older populations in Saudi Arabia poses significant challenges to the country’s healthcare system [14].

To encounter age-related health complications, physical activity (PA) is widely acknowledged as one of the vital approaches for older adults [15]. Furthermore, the effect of any type of physical activity may be varied not only by the type of physical activity itself but also by the style in which it is performed. A previously conducted study explored that group-based activities under the supervision of a professional expert were more beneficial than non-supervision programs for older adults. The supervised group-based program resulted in improvements in various health parameters that are crucial for older adults to maintain their overall well-being [16].

In addition, evidence suggests that participating in a group-based physical activity program is an effective approach to promoting persistent engagement in PA, particularly for older adults, and helps in maintaining an appropriate quality of life and mitigating the adverse consequences of social isolation [17]. Another study found that older adults benefited greatly from group exercise, or PA, in maintaining their physical, psychological, and social well-being. It also improved older adults’ overall health conditions and enhanced social interactions with their friends and peers, enabling them to enjoy their lives more [18]. As a form of PA, walking is often regarded as highly popular and widely recommended, especially for older adults and middle-aged people who may encounter challenges in performing other types of rigorous PA [19]. Evidence indicates that walking is also one of the most common forms of PA among Saudi Arabians [20].

Despite the beneficial effects of group-based physical activity intervention, the advantages of group-based intervention and the impact of professional supervision on the intervention are still not adequately examined, and more empirical research is required. This research gap was also uncovered by a recently conducted meta-analysis [21]. To address the aforementioned research gap, the present study aimed to investigate the comparative effects of supervised group-based walking intervention (SGBI) and non-supervised individual-based walking intervention (NSIBI) on frailty syndrome, cognitive skill or function, and quality of life among Saudi Arabian older adults who are not physically active. In addition, the current study also examined the comparative effects of SGBI and NSIBI on physical activity enjoyment and several health parameters, including body composition, blood pressure, resting heart rate, and overall walking performance, as secondary outcomes.

## 2. Materials and Methods

### 2.1. Study Design

The intervention was carried out from 15 September 2022 to 15 January 2023, as a 15-week walking intervention program with three 60 min sessions per week. This study applied a three-group design, which included two interventions and one control group. This study was conducted using a double-blinded (outcome assessors as well as data analysts) randomized controlled trial (RCT) to compare the effects of SGBI and NSIBI on frailty syndrome, cognitive function, and quality of life among inactive older persons in the KSA. In assigning interventions and control groups, the CONSORT procedure was followed [22]. The participants in the group-based intervention completed a walking intervention in groups under the supervision of a professional trainer. On the other hand, participants in the individual-based walking intervention completed the same intervention without the guidance of a professional trainer, whereas participants in the control group completed the intervention without receiving any training. This study procedure was completed following the international Good Clinical Practice Guidelines, and the protocol of this study was approved by the Research Ethics Committee (REC) of Hong Kong Baptist University (Ref: REC/20-21/0492) and the King Saud University, Saudi Arabia (Office for Human Research Protection (OHRP) with OHRP Institution Registration No.: IORG0006829, OHRP IRB Registration No.: IRB00008189, and IRB KACST Registration No.: H-01-R-002).

### 2.2. Sample Size

The sample size was determined with the utilization of G*Power software 3.1. The present study adopted a moderate effect size of 0.25 based on the previously conducted intervention studies, where the effect size ranged from moderate (0.25) to high (0.70) [23,24] and was the most frequently reported. In order to achieve a statistical power of 80% to detect a difference in treatment efficacy, with a threshold for significance of 5% for a two-sided test, a sample size of 35 participants for each group (a total of 105 for three groups) was anticipated to be recruited. Taking into account a 20% probable dropout rate, the present study required a total of 126 participants for 3 groups (42 individuals for each group) to complete two-way repeated measures at two points (pre- and post-test). Finally, 108 participants participated in the intervention, of whom 42 were in the SGBI, 36 were in the NSIBI, and 29 were in the control group. One participant left the intervention program for personal reasons, and 107 participants completed the whole intervention procedure. To conform to traditional norms in the KSA, males and females participated in the walking intervention in separate groups and were randomly and evenly divided into two intervention groups and a control group.

### 2.3. Participants Recruitment Procedure

Using the convenience-sampling technique, participants were recruited in the KSA according to the predetermined selection criteria. In the KSA, individuals under the age group 55–64 were more sedentary or inactive [25], and according to the United Nations, individuals’ minimum age considered for older adults is 60 years or above [3]. The targeted age group for the participants was 60–70 years old, including both males and females. Finally, the participants who were interested in participating in this study voluntarily and agreed to provide written consent were recruited through screening with the following eligibility criteria: (1) being aged 60–70 years; (2) having the ability to walk without any assistive device; (3) being in good health and living independently in their communities; (4) not having any cardiovascular or related ailments that would hinder intensive walking; (5) passing the PAR-Q screening or having a physician’s advice on readiness to participate in exercise training; (6) not having any diagnosed cognitive impairment; and (7) being sedentary or physically inactive. Being physically inactive means being an individual who has not met the World Health Organization’s recommended level of physical activity.

### 2.4. Grouping and Randomization

**Figure 1:**
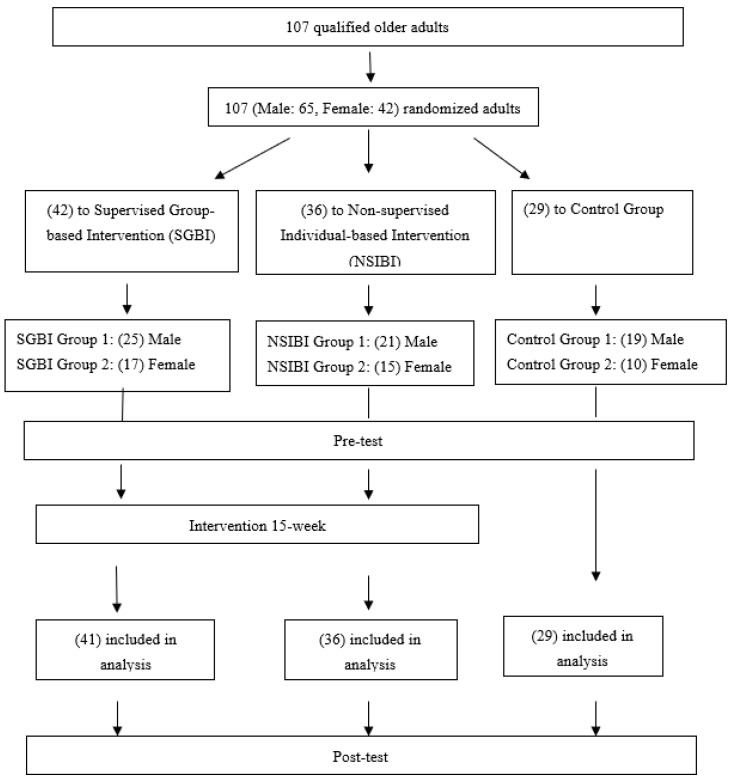
CONSORT Diagram for Participant Recruitment and Intervention.

By drawing lots, all the qualified participants, male (*n* = 65) and female (*n* = 42), who had signed the consent form were assigned randomly into three groups, which include: (1) SGBI (*n* = 42); (2) NSIBI (*n* = 36); and (3) Control Group (CG, *n* = 29). The assignment and data collection were carried out in accordance with the CONSORT process [22]. (Schulz et al., 2010) (Figure 1).

### 2.5. The Intervention

#### 2.5.1. Prescription for Walking Intervention

A 15-week walking intervention involving three sessions per week (50–70 min/session) was conducted in separate groups for males and females with the supervision of trained professionals or research assistants of the same gender as the participants. The ACSM concept of progressive training was adopted in the intervention program. The intensity and number of repetitions were increased according to the 4 levels (level 1: 1–4 weeks, level 2: 5–8 weeks, level 3: 9–12 weeks, and level 4: 12–15 weeks) (protocol is published) [26]. The progression levels could help to ensure that physically inactive participants have enough time to train with an adequate level of fitness, become accustomed to the regular walking schedule, and avoid musculoskeletal injuries as well as overtraining.

Furthermore, intensity level, number of steps, and anticipated heart rates were also included in the training sessions. Each participant was advised to wear a wrist-worn Fitbit Charge 3 (Fitbit Inc., San Francisco, CA, USA) to monitor the exercise intensity changes in heart rate with respect to their targeted heart rate zones and total steps after each session was completed. Individuality was also used as a training concept for the people who were walking, along with progression. They were allowed to reach their step goals considering their individual heart rates and their physical condition while walking. Please refer to [26] for the details of the intervention design and protocol.

Also, to support the culture of KSA, the walking intervention was conducted separately with men and women. In this set-up, there were four groups: [two groups (one male and one female) under SGBI and two groups (one male and one female) under NSIBI. Two professional fitness instructors (one male and one female for male and female groups, respectively) were assigned to the supervised group-based intervention programs to ensure that all participants received the proper direction and supervision. On the other hand, the NSIBI was conducted by a research assistant (male and female) to confirm attendance as well as general safety issues but did not offer professional advice or guidance. Only data from participants who completed a minimum of 70% of the training sessions during the 15-week walking treatments were included in the analysis.

#### 2.5.2. The Control Group

During the 15-week intervention program, the control group (CG) participants did not take part in any intervention, but they were required to maintain a daily record of their PA, illness, use of medications, and other health-related behaviors. The research assistant (RA) additionally asked CG participants to report any notable changes in the aforementioned aspects. RA checked daily records through a mobile phone or telephone communication every two weeks during the trial. Data from the participants who had modified their usual lifestyle (specifically engaging in regular PA) were excluded from the analysis.

### 2.6. Outcome Measures

#### 2.6.1. Primary Outcomes

##### Health-Related Quality of Life

A commonly used health survey instrument, the short form 36 (SF 36) health survey questionnaire, is widely used to assess older adults’ health-related quality of life (HRQoL) [27]. The SF-36 questionnaire comprises 36 items that cover various aspects of both physical and mental well-being [28]. The overall internal consistency of the questionnaire, as measured by Cronbach’s α coefficient, was reported to be 0.87 [29]. In the current study, the SF-36 was used to assess the quality of life of older adults [28].

##### Frailty

Frailty levels in older adults were examined with the use of a Physical Performance Test (PPT) involving several tasks. PPT has two versions, including a 9-item and a 7-item scale. In our study, we used the 9-item scale, and participants were instructed to complete nine standardized tasks included in the questionnaire. It took around 10 min to complete all the tasks for each participant. Each task scores 0–4, and 9 items score 36, with higher scores indicating better performance [30,31]. The Cronbach’s alpha and the internal reliability of the PPT are 0.87 and 0.99, respectively [30].

##### Cognitive Function

The cognitive status of older adults was examined using the Mini-mental State Examination (MMSE). It is an effective tool for screening the level of cognitive impairment at a certain point in time, observing cognitive changes over time, and assessing treatment response [32]. The reliability of the MMSE was acceptable, with a high Pearson *r* of 0.83 and a correlation for test-retest of 0.98 for older adults [32]. Each participant took a 10–15-min test that assessed memory, language, attention, orientation, and visuospatial skills. The MMSE scores range from 0 to 30; a score of 24 or higher is typically indicative of normal cognitive functioning or the absence of cognitive impairment. On the other hand, MMSE scores ranging from 0–17 and 18–23 indicate severe and mild impairment, respectively.

### 2.7. Secondary Outcomes

#### 2.7.1. Health Parameters—Body Composition, Resting Heart Rate, and Resting Blood Pressure

The effects of walking on participants’ several health parameters, including body composition, resting blood pressure, and resting heart rate, were also assessed. The participants’ body composition, which included the percentage of body fat and lean body mass, was measured by Tanita MC780U (Tanita Corporation, Tokyo, Japan, 2020). Evaluations of body composition using this model have been confirmed to exhibit a strong correlation with the Dual-Energy X-Ray Absorptiometry Scan (*p* < 0.001; r = 0.976; ICC [95% CI]: 0.95 [0.93–0.97], concordance coefficient: 0.955) [33]. In addition, participants’ body mass index (BMI) was also estimated by the RA through the measurement of participants’ heights and weights. The wrist-worn Fitbit Charge 3 was used to record participants’ resting heart rate. On the other hand, participants’ resting blood pressure was also recorded with the application of the Lenus Automatic Blood Pressure Monitor DP65 (MDF Instruments, California, USA). All the aforementioned assessments were carried out in the pre-test and post-test for comparison. The Intraclass Correlation Coefficients and Standard Error of Measurement for Fitbit Charge 3, in the context of heart rate and activity at rest, during a fitness test, and in recovery, range from 0.92 to 0.97 and 1.45 to 2.10, respectively [34].

#### 2.7.2. Walking Performance

The duration and the heart rate zones of each participant to complete the predetermined number of steps were measured in each intervention session to analyze performance and walking improvement. The data were recorded by the Fitbit Charge 3.

#### 2.7.3. Intervention Experience Evaluation

A few open-ended questions addressing the participant’s attitudes toward the intervention and their PA behavior after the intervention were asked at the post-test in order to further analyze and compare participants’ perceptions after the completion of the intervention.

### 2.8. Statistical Analysis

The demographic information and physical health characteristics such as gender, age, living conditions, body height, weight, resting heart rate, and blood pressure were described using descriptive statistics. The primary outcomes were analyzed via an Intention-To-Treat (ITT) approach, with a subsequent sensitivity analysis conducted on the available data. The Last Observation Carried Forward method was used to handle missing data. Per-protocol analysis of the available data were also used to investigate secondary outcomes. The threshold for statistical significance was set at *p* < 0.05. All data were processed using SPSS Version 26.0 (IBM, Chicago, IL, USA). A one-way repeated measures ANOVA was used for within-group tests to assess changes in each outcome parameter between the pretest and posttest. In cases where the sphericity assumption was violated, the Greenhouse-Geisser or Huynh-Feldt correction was applied based on the epsilon value. To investigate differences between pretest and posttest, a post hoc analysis with Bonferroni correction was performed. Mean differences along with 95% Confidence Intervals (CIs) were reported. For between-group comparisons, a one-way ANCOVA incorporating baseline value as a covariate was carried out at the posttest to evaluate the effect of group on each outcome parameter.

## 3. Results

### 3.1. Demographic Information

Table 1 illustrates the demographic information and the health parameters of the participants. Among the 107 participants, 65 were male and 42 were female, with a mean age of 62.53 years (SD = 4.95). The mean age of the participants for the SGBI, NSIBI, and control groups was 62.37 (SD = 4.89), 61.66 (SD = 3.49), and 64.18 (SD = 6.61). The mean percentage of participants’ fat content was lower among the participants in the control group, 38.8 (SD = 11.09), than that of the participants in the intervention groups, representing an almost similar percentage of fat content. In addition, other components, including muscle percentage, total body water, and visceral fat content, were found to be at almost similar levels for all groups. However, the amount of muscle mass (in kg) was also lower among the participants in the control group than those in the intervention groups (lower than about 13 kg and 14 kg from SGBI and NSIBI, respectively). In addition, the mean BMI values for the participants in the SGBI and control groups indicated overweight status; on the other hand, participants in the NSIBI were found to be obese individuals on the basis of their average BMI values.

### 3.2. Effect of Walking on Health-Related Quality of Life, Frailty, and Cognitive Functions

For the primary outcome, the level of health-related quality of life, frailty, and cognitive function among Saudi Arabian older adults in different intervention groups were measured before and after the intervention using a one-way repeated measure ANOVA. One-way analysis of covariance (ANCOVA) was conducted for the between-group test at the post-test, and the baseline value was used as a covariate. The data are normally distributed with all items’ z scores no greater than 3.3.

### 3.3. Within-Group and Between-Group Effects through Repeated Measure ANOVAs

Among the primary outcomes, participants in both intervention groups showed significant within-subject differences in the Physical Function subscale of the health-related quality of life scale only, with F(1,20) = 23.03, *p* < 0.001, and F(1,18) = 27.22, *p* < 0.001, respectively. Though the score decreased from the pre- to post-test in both the SGBI (−17.71%) and NSIBI (−26.57%), a greater decrease was found in the case of the NSIBI (Table 2 and Table 3). In addition, participants in the SGBI also showed significant within-subject differences in physical performance tests, with F(1,20) = 19.64, *p* < 0.001. However, no significant “Time X Group” interaction effect was found in all primary outcome measurements (Table 3). Among the secondary outcomes, no indicators were found to be significant within-subject as well as between groups (Table 4).

### 3.4. Between Group Effects

In the ANCOVA test, the Physical Performance Test revealed significant between-group differences in the post-test based on the baseline results, with F(2,51) = 9.21, *p* < 0.001. The pairwise comparisons demonstrated that SGBI participants performed significantly better than those of the NSIBI. At the posttest, the physical performance test score increased by 12.57% from the baseline among the participants in the SGBI group compared to the NSIBI group (2.40%). However, no significant between-group effects were found in other indicators, including the secondary outcome indicators.

### 3.5. Walking Performance

**Figure 2:**
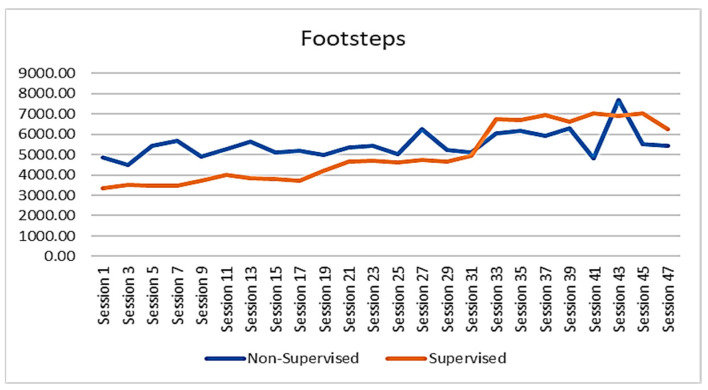
Changes in average footsteps count between SGBI and NSIB participants during the intervention (session by session).

**Figure 3:**
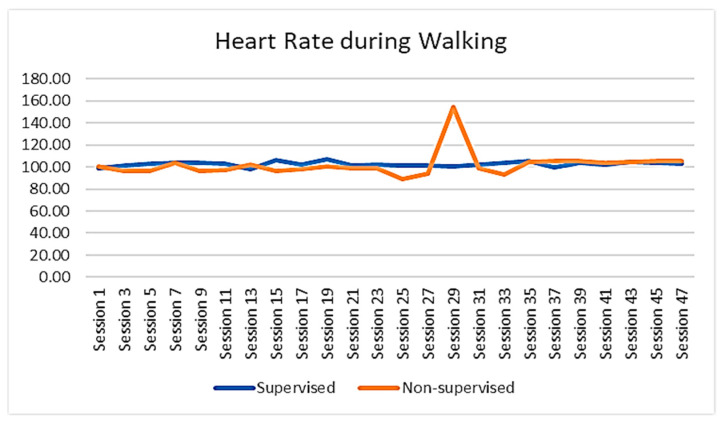
Changes in average heart rate while walking between SGBI and NSIB participants during the intervention (session by session).

**Figure 4:**
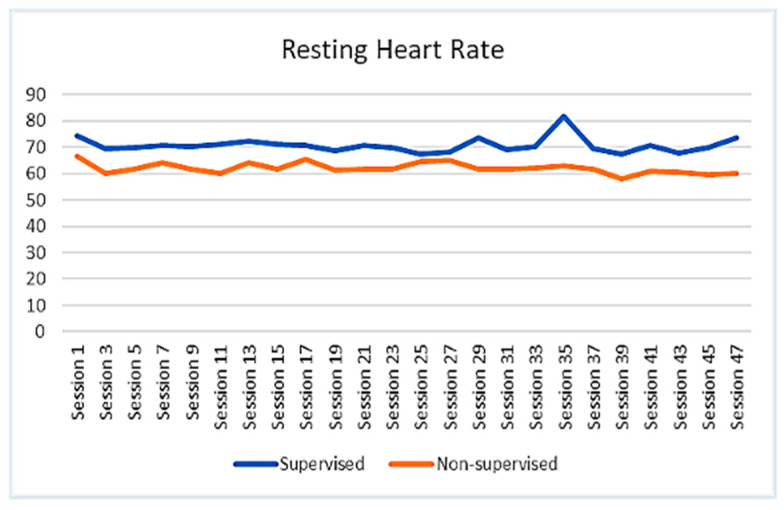
Trend of changes in average resting heart rate between SGBI and NSIB participants during the intervention (session by session).

The walking performance of the participants was recorded and extracted from the FITBIT devices. The average session footsteps, average session resting heart rate, and average session heart rate of the participants were demonstrated to show the trend of changes across the whole intervention period. The results revealed that the participants of SGBI had engaged in a progressive walking program and that their overall average number of steps had increased steadily from session to session, increasing from around 4000 steps per session to around 7000 steps per session. On the other hand, average footsteps for the participants of the non-supervised individual-based intervention were not steady throughout the intervention (Figure 2). However, the average heart rate during walking in the non-supervised intervention group did not show a noticeable change throughout the intervention period (Figure 3). It was also worth noting that the resting heart rate of both groups showed a slight declining trend throughout the intervention period (Figure 4). No data were available for the control group participants.

## 4. Discussion

The present study results demonstrated that among eight different subscales of the quality-of-life scale, the physical function subscale showed significant within-subject differences. Our study results demonstrated a significant difference in the post-test level of the physical performance test score, which is a measure of frailty among older adults. Participants in the SGBI group exhibited a greater increase in physical performance test scores than the participants in the NSIBI group. Frailty is the most significant age-related health condition, characterized by an impairment in the functioning of multiple physiological systems and a greater vulnerability to stressors. Frailty increases the likelihood of adverse consequences, including an increased risk of falls, the need for hospitalization, and mortality [35,36]. Previously conducted research results indicated that the situation of pre-frailty and frailty in the Kingdom of Saudi Arabia was 47.3% and 21.4%, respectively [37]. Evidence indicates that an increased level of physical performance is related to an increase in the intensity of physical activity, which could reduce the level of frailty [38]. Walking is considered the most preferred form of PA, particularly for older adults, and walking on a regular basis may confer significant health benefits in terms of improving physical performance and fitness as well as preventing the incidence of physical disability in older individuals [39,40].

Walking intervention showed a better result in the physical functioning domain of quality of life among the participants of SGBI than that of NSIBI. In both of the intervention groups, the physical function score decreased in the posttest, whereas NSIBI participants showed a higher rate of decrease, which is not aligned with previous research outcomes. Evidence revealed that physical function is the capability of an individual to perform activities of daily living, including both fundamental and instrumental [41], that reflect motor skills and control, usual PA, and physical fitness, and is also regarded as an important predictor of health complications, functional independence, incidence of disability, and mortality [42]. Previous study findings revealed a significant association between higher levels of physical function and the health-related quality of life among older adults. [42]. Evidence also revealed that older people with walking habits had greater levels of physical function. [43] Evidence also showed that older individuals who were physically active had a greater level of physical function and subsequent quality of life, and aging-related declines in physical function might affect the overall quality of life [44]. One of the possible reasons for the decline in physical function observed among the participants in the present study may be related to the natural consequences of the aging process. Evidence has revealed that the aging process may often be associated with natural degradation in almost all of the physiological systems of the human body, leading to lower performance in physical function, especially among older adults [45]. Yet, it is noteworthy that our participants had high BMI and low physical function levels during the baseline test and were less likely to participate in regular and high-intensity physical activity. Hence, the other possible reason for the decline in physical function observed among the participants may also be related to the sudden increase in their daily exercise intensity, which subsequently impacted their regular physical activities and functional abilities. This unexpected outcome also provided us with an insight into the need to reconsider the design of the intervention protocol, in particular to further evaluate the suitability of the walking program intensity for older adults who are less capable and physically inactive.

The present study results indicated that walking interventions increased older adults’ overall step count session by session. This might be due to the instructions and guidelines provided by the supervisor or coaches during the intervention. Moreover, under the supervision environment, participants showed a steady rising trend in their number of steps and steady heart rate intensity per session. This was in contrast to the fluctuating change among those in the non-supervised, individual-based walking intervention group. This indicates that a SGBI appears to have a more favourable outcome than that of a non-supervised, individual-based program. Therefore, the research outcomes supported the intervention design as a guideline for having an effect on the walking intervention program, while coaches, as the supervisors of the walking intervention, are allowed to modify the standard and goals of the walking program depending on the participants’ health conditions and physical ability. While the current walking program is also suitable for older adults who are less active and at a risk of obesity (BMI of 29 or above). This finding was aligned with a study conducted earlier by Harris et al. (2015) indicating that walking intervention increased the average daily step counts for the older adults in the intervention group compared to the control group [46]. Individuals’ daily step count is considered a simple as well as direct measure of the amount of PA and is also related to the time spent performing moderate-to-vigorous intensity of PA (MVPA). Evidence indicated that an increased daily step count was associated with improved bone and cardiovascular health as well as a reduced risk of mortality [47]. Evidence also suggests that for healthy older adults, regular engagement in 30 min of MVPA along with normal daily activities is equivalent to doing around 7000–10,000 steps per day [48]. In our study, walking intervention increased older adults’ daily step count from 4000 steps/day to 7000 steps, which was near the recommended number of steps for MVPA. This finding was inconsistent with a study carried out in Saudi Arabia indicating that the average daily step count for males suffering from chronic obstructive pulmonary diseases (COPD) was 4124 ± 2039 steps/day, which was lower than the recommended level [49].

In addition, our study indicated that resting heart rate for both of the intervention group participants was slightly in a declining state, and in the case of average heart rate, participants in the SGBI group showed almost non-noticeable change throughout the intervention, but NSIBI group participants showed different levels of average heart rate at different time periods of the intervention. Individuals’ heart rate is an important indicator that is extensively utilized in determining health status, especially overall cardiovascular health [50]. Evidence reveals that resting heart rate (RHR) is positively associated with cardiovascular mortality, and regular participation in physical activity or exercise plays a vital role in reducing RHR [51].

Evidence suggests that a face-to-face individual training program is an effective method for modifying individuals’ attitudes that helps to increase the amount of physical activity [52]. Evidence also suggests that supervised exercise programs tend to be more effective, particularly for older individuals, because professional trainers can monitor the intensity of the activity and ensure that participants fulfill their targeted level of physical activity. Better participation in supervised walking can help older adults maintain their better level of cardiopulmonary functions, and the probable reason might be the fact that older adults in the supervised group usually spend more time performing their walking activities at moderate to higher levels of intensity than the older adults monitoring their activities by themselves. Moreover, study results also suggest that, besides supervised walking, self-monitored walking also has benefits for maintaining continuity in walking due to their autonomy to modify their walking habits by their own decision [53].

### 4.1. Strength and Practical Application of Findings

It is recognized that research related to walking in Saudi Arabia has primarily been cross-sectional and focused on environmental development. Researchers have typically relied on questionnaires and geographical applications, with these studies indicating that 80% of participants enjoyed walking due to the accessibility of walking routes on average. The studies also suggested that enhancing sidewalk walkability could potentially boost pedestrian activity. However, the region’s arid climate and high temperatures may act as deterrents to walking [54,55]. It was also discovered that elderly individuals with chronic illnesses in Saudi Arabia tend to engage less in walking-related activities, highlighting the need for increased attention in this area. Physical therapists have also employed walking-based therapeutic methods with stroke patients [49,56]. As such, this study offers valuable insights into walking interventions and emphasizes the significance of group and social elements in walking activities. The findings would not only aid in understanding the differing effects of SGBI and NSIBI, but they will also help raise awareness among government bodies and the public in Saudi Arabia regarding issues of aging and strategies to enhance intervention effectiveness. Armed with this evidence, the government, universities, and relevant NGOs in Saudi Arabia can justify the formulation of guidelines and policies and, crucially, the allocation of more resources towards promoting healthy and active aging within the country. To tackle this issue, we suggest the following recommendations:

Firstly, develop and implement public policies that promote walking and active aging. The Saudi Arabian government should work closely with the Ministry of Health, the Ministry of Sports, and other relevant bodies to establish guidelines on walking and other active aging activities such as jogging and home-based aerobic exercises. The Ministry of Health and Ministry of Sports should offer state-subsidized programs to allow older adults in Saudi Arabia to engage in low-cost active aging programs.

Secondly, the Saudi Arabian government should establish or develop infrastructure for safe walking, especially in places near housing estates. The government should create safe walking infrastructure that is accessible and user-friendly for older adults, including pathways, sidewalks, and pedestrian bridges that are not restricted to sports complexes or parks.

Thirdly, the Saudi Arabian government should provide more financial resources and support to senior centers and care homes, offering active aging programs such as physical therapy, rehabilitation services, and fitness coach training sessions, enabling older adults to remain active and healthy.

Finally, the Ministry of Health and Ministry of Sports should encourage non-governmental organizations to offer intergenerational activities such as a buddy walking program that pairs older adults with a young adult jogger, and the teenage or young adults’ jogger is required to lead and perform exercise together with the older adults. These activities will provide opportunities for older adults to engage with other age groups, increase community and social connections, and improve their physical, mental, and social well-being.

Implementing these recommendations will promote walking as well as active aging among the older adults in Saudi Arabia, resulting in healthier, more active lifestyles for this population.

### 4.2. Limitations

There are several limitations to our study. Firstly, due to the change in regulation of the recruited elderly center, the post-test data for the control group were not possible to collect. For this reason, it was not possible to perform a comparison regarding the effects of walking on different health indicators among the three groups of participants. It might affect the quality of this study and weaken the rigor of the findings. Secondly, participants could not be randomized equally in all groups, including two interventions and one control group, due to their disagreement to provide written consent to participate in this study. Thirdly, follow-up observation after the completion of the 15-week intervention was not possible due to time constraints, which affected the results to depict a comparative scenario of the effects of walking on different health indicators, which include frailty, quality of life, and cognitive functions, for older adults at 3-time points (pre-, post-, and follow-up tests). Fourthly, male participants were more than females, and for this reason, equal representation of males and females in the same group was not possible.

## 5. Conclusions

Older adults are highly susceptible to age-related complications that may cause frailty and impairment in cognitive functions and reduce the overall quality of life. Physical activity, particularly walking, has enormous health benefits, especially for older adults’ health and overall well-being. The current study explored whether walking interventions had a significant impact on a physical performance test that measures the level of frailty. In addition, the average step count was also increased session by session in the case of the intervention groups that would benefit older adults in Saudi Arabia by encouraging them to walk at an appropriate level. Therefore, an appropriate walkable environment and counseling regarding the benefits of regular walking would be an effective approach to maintaining frailty-free, improved cognitive function, and a better quality of life, particularly for the older adults of the Kingdom of Saudi Arabia. In the future, more RCT should be conducted to assess the effectiveness of walking on other parameters that have an influential effect on maintaining healthy and active aging.

## Figures and Tables

**Table 1:** Demographic Information and Health Parameters (*n* = 107).

	SGBI	NSIBI	CG
	Mean (SD)	Mean (SD)	Mean (SD)
**Age (years)**	62.37(4.89)	61.66 (3.49)	64.18 (6.61)
**Fat Percentage (%)**	40.13 (10.39)	41.38 (9.72)	38.8 (11.09)
**Muscle Percentage (%)**	56.59 (9.95)	55.41 (9.34)	57.83 (10.61)
**Muscle Mass (kg)**	44.1 (9.74)	45.12 (9.54)	31.12 (4.05)
**Total Body Water (kg)**	33.31 (5.83)	34.52 (5.33)	31.23 (4.05)
**Visceral Fat (level)**	14.84 (4.77)	15.97 (3.76)	14.80 (4.80)
**Body Mass Index (kg/m^2^)**	29.96(5.77)	31.03(3.65)	29.1 (4.86)

kg = kilogram; SD = Standard deviation; BMI is measured as kg/m^2^.

**Table 2:** Descriptive Statistics of the Primary Outcome.

		SGBI		NSIBI		CG	
		Baseline	Post	Baseline	Post	Baseline	Post
**Frailty**	**Physical Performance**	22.67 (5.00)	25.52 (2.91)	22.95 (4.55)	23.50 (3.75)	20 (4.69)	/
**Quality of Life**	**Physical Function**	25.80 (4.33)	21.23 (3.32)	26.35 (4.156)	19.35 (5.33)	28.25 (3.5)	/
	**Role Physical**	17.29 (3.78)	17.09 (3.78)	18.2 (2.09)	18.2 (2.09)	18.25 (1.71)	/
	**Body Pain**	8.86 (1.98)	9.21 (1.80)	7.99 (2.75)	8.65 (2.36)	9.68 (1.29)	/
	**General Health**	22.24 (2.21)	22.24 (2.4)	21.65 (3.76)	21.75 (0.71)	23 (1.41)	/
	**Vitality**	17.61 (2.85)	18.86 (2.39)	17.3 (3.54)	18.2 (3.10)	19.5 (2.08)	/
	**Social Functioning**	8.62 (1.68)	8.81 (1.60)	8.75 (2.25)	8.5 (1.76)	9.25 (1.5)	/
	**Role Emotional**	12.90 (2.32)	13.67 (1.98)	13.15 (3.01)	12.75 (2.59)	13.75 (1.26)	/
	**Mental Health**	23.04 (3.88)	24.81 (2.80)	23.45 (4.17)	24.3 (2.9)	25.75 (1.5)	/
**Cognitive Function**	**Mini-mental state examination**	29.05 (1.86)	29.62 (1.12)	28.13 (2.80)	28.6 (1.69)	26.75 (3.30)	/

Note: Mean (SD). Post-test data for the control group participants were absent due to the change in elderly center regulation, and they were excluded from the analysis.

**Table 3:** Within-subject Effect of the Primary Outcome.

		SGBI			NSIBI		
		df	F	Partial η2	df	F	Partial η2
**Frailty**	**Physical Performance**	1	19.64 ***	0.495	1	0.852	0.043
**Quality of Life**	**Physical Function**	1	23.03 ***	0.54	1	27.22 ***	0.59
	**Role Physical**	1	0.18	0.009	1	0.00	0.00
	**Body Pain**	1	0.97	0.046	1	1.219	0.06
	**General Health**	1	0.00	0.00	1	0.014	0.001
	**Vitality**	1	4.88 *	0.196	1	2.23	0.105
	**Social Functioning**	1	0.18	0.009	1	0.198	0.01
	**Role Emotional**	1	1.54	0.072	1	0.325	0.017
	**Mental Health**	1	4.59 *	0.187	1	0.81	0.041

* *p* < 0.05, *** *p* < 0.001.

**Table 4:** Descriptive Statistics of the Secondary Outcomes.

	SGBI		NSIBI		CG	
	Baseline	Post	Baseline	Post	Baseline	Post
**Body Mass Index**	29.96 (5.77)	30.25 (5.33)	31.03 (3.65)	31.02 (3.82)	28.84 (4.24)	/
**Resting Heart Rate**	79.27 (10.1)	81.48 (10.01)	75.43 (10.48)	76.71 (9.87)	78.60 (7.23)	/
**Resting Blood Pressure (Upper)**	128.64 (20.68)	125.91 (18.33)	120.70 (17.81)	127.09 (14.22)	/	/
**Resting Blood Pressure (Lower)**	83.06 (19.38)	85.61 (17.43)	80.74 (8.27)	80.87 (6.43)	/	/
**Physical Activity Enjoyment**	/	4.15 (0.62)	/	4.32 (0.52)	/	/

Note: Mean (SD). Post-test data for the control group participants were absent due to the change in elderly center regulation, and they were excluded from the analysis.

## Data Availability

The data presented in this study are available upon request from the corresponding author. The data are not publicly available to ensure confidentiality of study participants.

## Abbreviations

WHO	World Health Organization
KSA	Kingdom of Saudi Arabia
PACES	Physical Activity Enjoyment Scale
HRQoL	Health-related quality of life
PPT	Physical Performance Test
MMSE	The Mini-mental state examination
ANOVA	One-way repeated measure analysis of variance
ANCOVA	One-way analysis of covariance
